# Reconsidering inequalities in COVID-19 vaccine uptake in Germany: a spatiotemporal analysis combining individual educational level and area-level socioeconomic deprivation

**DOI:** 10.1038/s41598-024-75273-9

**Published:** 2024-10-13

**Authors:** Marvin Reis, Niels Michalski, Susanne Bartig, Elisa Wulkotte, Christina Poethko-Müller, Daniel Graeber, Angelika Schaffrath Rosario, Claudia Hövener, Jens Hoebel

**Affiliations:** 1 Department of Epidemiology and Health Monitoring, Robert Koch Institute, Nordufer 20, Berlin, 13353 Germany; 2 Department of Sociology, London School of Economics, London, United Kingdom; 3 Department of Sociology, Freie Universität Berlin, Berlin, Germany; 4 Department of Infectious Disease Epidemiology, Robert Koch Institute, Berlin, Germany; 5 Socio-Economic Panel, German Institute for Economic Research, Berlin, Germany; 6 IZA Institute of Labor Economics, Bonn, Germany; 7 Center for Economic Policy Analysis, University of Potsdam, Potsdam, Germany

**Keywords:** COVID-19, Vaccine uptake, Socioeconomic factors, Social Deprivation, Diffusion of Innovation, Regional disparities, Infectious diseases, Disease prevention, Health care economics, Health policy, Public health, Epidemiology

## Abstract

Combining the frameworks of fundamental causes theory and diffusion of innovation, scholars had anticipated a delayed COVID-19 vaccination uptake for people in lower socioeconomic position depending on the socioeconomic context. We qualify these propositions and analyze educational differences in COVID-19 vaccination status over the first ten months of Germany’s vaccination campaign in 2021. Data from the study “Corona Monitoring Nationwide” (RKI-SOEP-2), collected between November 2021 and February 2022, is linked with district-level data of the German Index of Socioeconomic Deprivation (GISD). We estimated the proportion of people with at least one vaccination dose stratified by educational groups and within different settings of regional socioeconomic deprivation at three time points. Logistic multilevel regression models were applied to adjust for multiple covariates and to test cross-level-interactions between educational levels and levels of area-level socioeconomic deprivation. Vaccination rates were lower among respondents with lower education. With increasing area-level socioeconomic deprivation, educational differences were larger due to particularly low vaccination rates in groups with low education levels. The analysis of vaccination timing reveals that educational gaps and gaps by area-level socioeconomic deprivation had appeared early in the vaccination campaign and did not close completely before the 4th wave of COVID-19 infections

**Supplementary Information:**

The online version contains supplementary material available at 10.1038/s41598-024-75273-9.

## Introduction

During the pandemic waves of SARS-CoV-2 infections in Germany since March 2020, people with fewer resources, poorer living and working conditions, and those in deprived areas faced a higher risk of infection and severe or fatal progression of coronavirus disease 2019 (COVID-19)^1–4^. The availability of vaccines was an eagerly awaited remedy to mitigate the perils of the pandemic and relieve people from containment measures. Widespread access to free vaccination was expected to mitigate socioeconomic inequalities in risks of infections and severe progression of the disease across the population.

In Germany, as in most other countries, vaccine doses were limited when they became available in late December 2020. To protect those at high risk of severe disease, the German vaccination campaign followed a prioritization scheme. Older adults, individuals with preexisting conditions (e.g., cardiovascular diseases, diabetes, chronic illnesses), and those employed in high-exposure occupations (e.g., nursing staff and essential workers) were eligible for early COVID-19 vaccination^5^. After the removal of the prioritization scheme on June 7, 2021, vaccination was available for all residents aged 18 and older. Generally, the prioritization scheme was one of few structural barriers to vaccination. Otherwise, vaccination was free of charge and provided at temporary vaccination sites throughout 2021, and it was not compulsory for any social group during the period covered by this study. From April 7, 2021, primary healthcare providers were also allowed to vaccinate, further improving access.

By early November, after ten months of the vaccination campaign, almost 80% of the German adult population had acquired basic immunization through two vaccine doses^6^. With only about 10% estimated to have had prior SARS-CoV-2 infections before the 4th wave, vaccination significantly contributed to population immunity^6^. Despite these achievements, Germany’s vaccination rates remained average among European countries and were not high enough to extensively mitigate hospitalizations and fatalities during the fourth wave of infections in Fall 2021^7^.

Survey-based studies on COVID-19 vaccine uptake in Germany have found lower vaccination rates among less educated individuals and among those with lower incomes^8,9^. These disparities were also observed in other high-income countries^10,11^. Analyses of the spatial distribution of vaccine uptake showed considerable variation which was correlated with area-level socioeconomic indicators. Accordingly, register-based area-level analyses in the UK^11,12^ and the US^13^, as well as survey-based analyses in Germany^14^ found that vaccine uptake was lower in areas with high socioeconomic deprivation compared to affluent ones.

These findings raise the question of whether area-level socioeconomic differences merely reflect aggregated individual-level disparities or if the socioeconomic context exerts an additional independent influence. Moreover, the socioeconomic context may exacerbate disparities in vaccine uptake potentially resulting in particularly low vaccination rates among individuals with lower socioeconomic position in highly deprived areas. To date, little is known about how socioeconomic differences in vaccine uptake vary across regions and area-level socioeconomic characteristics. This study aims to address this gap and will also examine the timing of vaccination.

Prior research on vaccine uptake for diseases such as measles or influenza in Germany has been inconclusive with respect to socioeconomic differences^15–17^. However, the urgency of the COVID-19 pandemic may have exacerbated socioeconomic inequalities due to the varying pace of vaccination among different social groups. Socioeconomic differences in the timing of vaccination are often overlooked, despite their significant impact on reducing health risks. The novelty of the pathogen, affecting an immunologically naïve global population, and its rapid airborne spread required a swift adoption of necessary health prevention measures, most prominently vaccination.

Previous studies on socioeconomic inequalities in vaccination have mainly neglected the timing of vaccination. Although inequalities in vaccination coverage persisted after prioritization, socioeconomic factors likely had a greater impact earlier on. Examining the temporal dynamics of these inequalities in vaccination against a novel pathogen will provide deeper insights into socioeconomic differences in vaccine uptake in Germany.

Concerns about the temporal dynamics of vaccination were raised early in the pandemic by Rydland, et al.^18^. They used a framework combining fundamental cause theory (FCT)^19,20^ and DOI^21,22^ to predict the temporal dynamics and implications of socioeconomic inequalities in vaccine uptake during the early stages of the pandemic. They suggested a delayed vaccine uptake among the socioeconomically disadvantaged. FCT posits that inequalities in socioeconomic position are the underlying reason for health inequalities, as socioeconomic position dictates the access to resources, education, jobs, etc.^19,20^. Through these resources harmful circumstances are more likely to be avoided, a healthier life style can be purchased and beneficial health behavior is adopted. Education plays a major role in health prevention behavior ^20^. DOI theory captures the temporal aspects of adopting disease prevention and health protection behaviors^21,22^. It posits that the cumulative share of adopted health innovations over time follows an S-shaped curve: a few individuals adopt early, followed by a rapid increase until a peak, after which the rate of new adoptions slows and eventually stops, leaving some individuals who never adopt the innovation^21^. In combination with FCT, DOI theory suggests that individuals with higher socioeconomic positions—who possess more flexible resources such as knowledge, money, power, prestige, and beneficial social connections—have access to more diverse information sources and greater means to research and implement innovations early, leading to faster adoption^22^.

The spread of innovation also depends on the social context and networks. According to DOI, peer-to-peer communication among individuals with similar socioeconomic status leads to more effective information exchange and higher adoption rates of health innovations^21,22^. Consequently, homogeneous communities with many people having access to flexible resources might achieve collective goals, such as herd immunity through vaccination, more effectively. On the downside, peer-to-peer communication can also facilitate the spread of misinformation. However, individuals with more flexible resources are better equipped to resist misinformation. Given the observed area-level variations in vaccine uptake^23^, it is likely that areas with fewer flexible resources would exhibit greater socioeconomic differences in vaccination rates. This is because such contexts may not provide sufficient flexible resources, such as effective information exchange, to convince more people with lower socioeconomic resources to vaccinate.

Although the theoretical framework suggests mechanisms for contextual and network effects, these mechanisms are very difficult to assess empirically, and different mechanisms may simultaneously be at work, making them difficult to disentangle. Rather than explicitly testing the implications of DOI and FCT, our study is primarily descriptive, using the propositions about socioeconomic differences in the temporal dynamics of vaccine uptake within socioeconomic contexts as inspiration. We assume that the level of socioeconomic deprivation in German districts serves as a good proxy for measuring the average level of resources available within a socioeconomic context. Districts are a key political-administrative level with significant socioeconomic variation across Germany. They also served as the lowest administrative divisions for tracking and managing the COVID-19 vaccination campaign.

Socioeconomic deprivation is a common concept for the relative disadvantage attached to spatial entities^24,25^. We follow Townsend in his definition of deprivation “[as] a state of observable and demonstrable disadvantage relative to the local community or the wider society or nation to which an individual, family or group belongs”^25^. The ecological perspective suggests that individuals in deprived areas are not necessarily socioeconomically deprived themselves - as socioeconomically disadvantaged individuals also reside in affluent areas. However, the socioeconomic conditions in an area gradually impact individual economic opportunities, health behavior, and health outcomes^25,26^ in analogy to the propositions by Rydland et al.^18^.

The main focus of our study is to analyze whether educational differences in vaccine uptake depend on the area-level socioeconomic context and how large these gaps are. We expect more deprived districts to exhibit larger educational gaps in vaccine uptake. Additionally, we examine how educational differences in COVID-19 vaccination evolved over the course of the vaccination campaign. We provide a table outlining the key phases of the vaccination campaign in Germany, as well as the pandemic phases during the period under investigation, in the supplement (Supplementary Table A). We anticipate that the distribution of vaccinated individuals in the early stages was strongly influenced by the prioritization scheme, which remained in effect until June 7, 2021. During this period, socioeconomic inequalities in vaccine uptake were expected to be smaller. By August 2021, we expect that the majority of individuals inclined toward vaccination would have already been vaccinated, but those with lower education and those living in more deprived districts would have been slower, resulting in lower vaccination rates. From mid-August to the start of the fieldwork for the data we are using, additional motivations—such as restrictions for unvaccinated individuals and rising incidence and COVID-19-related mortality rates—could have convinced more people to get vaccinated, potentially narrowing the socioeconomic gaps.

## Materials and methods

### Data

We used data from the second wave of the “Corona Monitoring Nationwide” (RKI-SOEP-2) study and data from the German Socio-Economic Panel (GSOEP)^27^. The GSOEP is one of the world’s longest running nationwide, longitudinal population-based cohort studies with annual questionnaires. For RKI-SOEP-2, all GSOEP household members aged 14 and older in the gross sample were invited to participate in an additional survey in 2021, which included biospecimen and questionnaire data collection. The GSOEP and RKI-SOEP-2 data collection were carried out in accordance with the Declaration of Helsinki. Approval was obtained from the ethics committee of the Berlin Medical Association (reference ID Eth-33/20 as of September 21, 2021). All participants provided informed consent. All methods were performed in accordance with the relevant guidelines and regulations. The field phase ran from November 2021 to March 2022. A total of 11,162 subjects participated. The response rate was 53.7% according to AAPOR^28^ standard. For the present analysis, only the population aged 18 and older was considered (*N* = 10,288). The dataset includes weights to consider the selection processes for participation in GSOEP and RKI-SOEP-2 and adjust the sample to match official German population distributions concerning age, gender and the spatial distribution across federal states. More details on the study design, data collection and methods are described in Bartig, et al.^27^.

## Dependent variables: vaccination status at different time points

Vaccination status was determined by the respondents’ self-reports of having received at least one vaccine dose against COVID-19. In the survey, participants were asked about the number of vaccine doses they had received as well as the respective vaccination date and the type of vaccine. We used the vaccination date to estimate the weekly vaccination rates for our sample by calculating the proportions of respondents who had received at least one vaccine dose for each week. Additionally, we chose three pivotal time points of the German vaccination campaign for which the socioeconomic differences in vaccine uptake were estimated in the empirical analysis. The time points are described as follows:

The removal of the prioritization scheme on June 7, 2021 was the first pivotal time point. Until then, vaccination rates were expected to be significantly influenced by this scheme. For the second time point, August 16, 2021 was chosen. This date allowed for a sufficient ten-week period after the removal of the prioritization scheme, providing ample time for scheduling appointments and receiving vaccines. It was presumed that by this date, a majority of those willing to vaccinate had already received their first dose, as almost two-thirds of Germany’s adult population had been vaccinated at least once by then^23^. However, the vaccination campaign’s momentum had already begun to plateau, coinciding with a resurgence in COVID-19 incidence rates. On August 23, 2021 new restrictions were imposed, limiting access to public spaces (e.g. restaurants, events or hospitals) exclusively to vaccinated, recovered, or tested individuals (‘3G rule’).

The initiation of fieldwork on November 8, 2021 marks the third date under investigation. For subsequent dates following the first respondents’ completed questionnaires, data on vaccination status and dates were inconsistent, as some individuals may have been vaccinated in the interim. This incompleteness could potentially introduce selection bias. Furthermore, November 8, 2021 marked a time point when the COVID-19 incidence and associated mortality rates of the 4th pandemic wave had already reached unprecedented heights^7^. The period from August to November 2021 marked a critical phase for preventing infections, hospitalizations, and deaths in Germany. During this time, the unvaccinated population likely drove the exponential growth of infections, as evidenced by estimates from Maier, et al.^7^. About 61-76% of the effective reproduction numbers were attributed to this demographic during the country’s 4th wave of SARS-CoV-2 infections.

## Independent variables

The main independent variables were the participants’ educational levels (as a marker of individual socioeconomic position (SEP)) and socioeconomic deprivation of the respondents’ residential districts. Educational attainment, taken from the GSOEP 2020 wave and based on the 2011 International Standard Classification of Education (ISCED), was condensed into three categories: *low* (ISCED levels 0 – “Early childhood education”, 1 – “Primary education”, 2 – “Lower secondary education”), *medium* (ISCED levels 3 – “Upper secondary education”, 4 – “Post-secondary non-tertiary education”) and *high education* (ISCED levels 5 – “Short-cycle tertiary education”, 6 – “Bachelor’s or equivalent level”, 7 – “Master’s or equivalent level”, 8 – “Doctoral or equivalent level”).

To measure area-level socioeconomic deprivation, the most recent version of the German Index of Socioeconomic Deprivation (GISD)^29,30^ was linked to the individual data through district-level identifiers of the residential address. The GISD maps the socioeconomic deprivation of administrative German regions measured by area-level indicators of three main dimensions: education (e.g. proportion of employees without a professional qualification), employment (e.g. unemployment rate) and income (e.g. average net household income). Factor scores for each of the three dimensions are obtained from principal component analyses. They are normalized and additively combined with equal weights. The GISD provides a deprivation measure at various administrative levels. District-level scores are sorted into quintiles condensed into three categories: *low deprivation* (1st quintile), *moderate deprivation* (2nd to 4th quintile) and *high deprivation* (5th quintile).

The official districts of Germany were chosen as the contextual-level spatial unit for the analysis. Germany had 401 districts, comprising rural districts (*Landkreise)* and major cities (*Stadtkreise)*. They represent a spatial unit at the intermediate level of administration between federal states (Bundesländer) and municipalities (Gemeinden). Districts also served as the lowest administrative divisions for tracking and managing the COVID-19 vaccination campaign.

## Analysis strategy

The empirical analysis is presented in three parts. First, we estimate the vaccination rates by educational level and GISD category as of November 8. Second, we examine the temporal dynamics of vaccine uptake by socioeconomic group using survival analysis^31,32^. Self-reported vaccination dates were translated into calendar weeks, and reverse Kaplan‒Meier survival estimates^33^ were calculated by educational level and GISD category with results graphically depicted. The estimates detail the temporal dynamics of socioeconomic differences. Third, we use multilevel logistic regression models with random intercepts for German districts to analyze vaccination status at the three time points, accounting for clustering within districts^34^. We investigate whether socioeconomic differences in vaccine uptake remain substantial when individual and area-level variables are added simultaneously. In line with the general descriptive aims of our study, we use a minimal set of control variables at the individual level: age, sex and migration history. We also add preexisting medical conditions to adjust educational gaps for prioritized access to vaccination. And we control for known previous SARS-CoV-2 infection (if prior to the respective time point), since previous infection was considered equivalent to one vaccination dose to claim vaccination status and people previously infected were advised to wait several months before receiving vaccination. Cross-level interaction terms are included to test whether educational differences varied by the level of socioeconomic deprivation. All models are estimated using robust standard errors for clustered data^34^. GISD categories and a variable indicating residency in East or West Germany are added as context-level variables to account for historical differences between these regions. Predicted probabilities are calculated and graphically presented, based on models with the cross-level interaction of GISD and educational level. Weights adjust to consider the participation selection processes and to match German population distributions.

**Table 1:** Characteristics of the study population (unweighted).

Variables	*N*	Proportion in %
**Vaccination status at prioritization removal**		
No vaccination	3,073	29.4
One or more vaccinations	7,197	68.9
Missing	178	1.7
**Vaccination status at 3G rule**		
No vaccination	1,049	10.0
One or more vaccinations	9,221	88.3
Missing	178	1.7
**Vaccination status at start of fieldwork**		
No vaccination	704	6.7
One or more vaccinations	9,566	91.6
Missing	178	1.7
**Age Group**		
18–29	1,319	12.6
30–44	2,104	20.1
45–59	3,461	33.1
60–79	3,106	29.7
80+	458	4.4
**Sex**		
Male	4,804	46.0
Female	5,644	54.0
**Education**		
Low Education	967	9.3
Medium Education	4,948	47.4
High Education	4,061	38.9
Missing	472	4.5
**Area-level Deprivation (GISD categories)**		
Low Deprivation	2,512	24.0
Moderate Deprivation	6,335	60.8
High Deprivation	1,479	14.2
Missing	102	1.0
**Migration History**		
No migration history	8,750	83.7
Direct migration history	1,120	10.7
Indirect migration history	511	4.9
Missing	67	0.6
**Chronical diseases**		
None	4,876	46.7
One preexisting condition	2,807	26.9
Two or more preexisting conditions	2,765	26.5
**Previous infection before removal of prioritization scheme**		
No Infection	9,732	93.5
Previous SARS-CoV-2 Infection	475	4.6
Missing	202	1.9
**Previous infection before 3G rule**		
No Infection	9,719	93.4
Previous SARS-CoV-2 Infection	488	4.7
Missing	202	1.9
**Previous infection before survey start**		
No Infection	9,620	92.4
Previous SARS-CoV-2 infection	587	5.6
Missing	202	1.9
**Survey Region**		
West Germany	7,914	75.7
East Germany (incl. Berlin)	2,534	24.3

Conventional p values and Holm-Bonferroni adjusted p values^35^ are reported to evaluate statistical significance of pairwise differences in proportions and predicted probabilities. The subscript of the p values, e.g. p_36_, denotes the number of simultaneous tests, which is corrected for by the Holm-Bonferroni method if more than three groups are compared simultaneously. The sample characteristics are reported in Table 1 and Supplementary Table A provides an overview of all variables used in the empirical analyses and indicates their underlying concept and respective definitions. The statistical analyses were conducted using StataCorp LLC’s Stata Statistical Software: Release 17^36^.

## Results

### Descriptive results

Table 2 presents the proportions of individuals with at least one vaccine dose before the start of the survey stratified by education level and area-level socioeconomic deprivation. The rates show that vaccine uptake was approximately 95% among highly educated individuals, irrespective of area-level socioeconomic deprivation. Vaccine rates were also high across all educational levels within districts with low area-level socioeconomic deprivation. For individuals with medium education, uptake dropped from 94.9% in low deprivation areas to below 92% in moderate and high deprivation areas. Among those with low education, rates fell from 96.3% in low deprivation areas to 86.5% in moderately deprived areas and further to 82.3% in highly deprived areas. Differences in vaccination rates within low deprivation areas and among the highly educated were small and not statistically significant at p_36_ < 0.05. However, vaccination rates for low-educated individuals in highly deprived areas were significantly lower than those for low- and medium-educated individuals in low deprivation areas (Odds-Ratios OR = 0.18, p_1_ = 0.001; p_36_ = 0.034 and OR = 0.25, p_1_ = 0.002; p_36_ = 0.036) and also lower than those for highly educated individuals in highly deprived areas (OR = 0.23, p_1_ = 0.001; p_36_ = 0.035). Furthermore, vaccination rates for low educated individuals in moderately deprived areas were significantly lower than the rates in low deprivation areas regardless of the educational level areas (OR < 0.38, p_1_ < 0.001; p_36_ = 0.033).

**Table 2:** Vaccination rates by area-level socioeconomic deprivation and educational level with 95% confidence intervals at the field start (weights applied), N – number of observations (unweighted).

	Low Deprivation	Moderate Deprivation	High Deprivation	Total
Low Education	96.3 [92.8–98.1] *N* = 193	86.5 [80.5–90.9] *N* = 564	82.3 [68.2–90.9] *N* = 140	87.9 [83.5–91.2] *N* = 897
Medium Education	94.9 [92.8–96.4] *N* = 1,065	91.9 [90.3–93.3] *N* = 3,011	91.0 [87.4–93.6] *N* = 768	92.5 [91.3–93.5] *N* = 4,844
High Education	94.9 [92.2–96.7] *N* = 1,101	94.5 [92.7–95.8] *N* = 2,404	95.2 [91.8–97.2] *N* = 495	94.7 [93.4–95.7] *N* = 4,000
Total	95.0 [93.5–96.2] *N* = 2,359	92.3 [91.0–93.4] *N* = 5,979	91.1 [87.9–93.5] *N* = 1,403	92.9 [91.9–93.7] *N* = 9,741

## Survival analysis

**Fig. 1:**
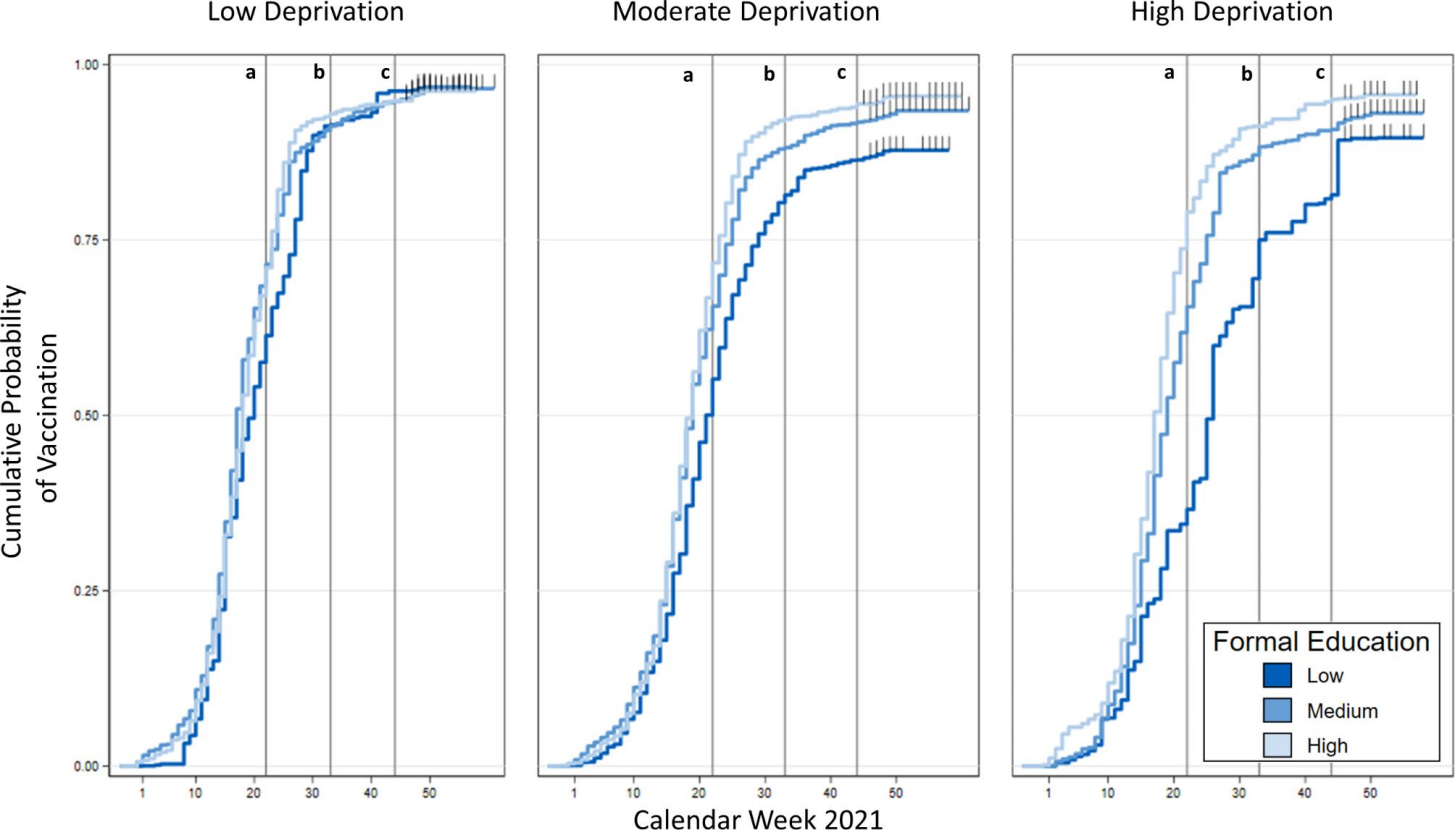
Education-specific reverse Kaplan—Meier survival curves for first vaccination by area-level socioeconomic deprivation over calendar weeks in 2021 (**a** – Prioritization removal, **b** – 3G Rule, **c** – Field start).

In the second part of the analysis, we examine the development of educational differences in vaccine uptake over the vaccination campaign. We present reverse Kaplan‒Meier survival curves by educational level for the three deprivation categories across calendar weeks starting with the first vaccination in our sample (Fig. 1).

The reverse Kaplan‒Meier survival curves illustrate that, prior to the prioritization scheme (week 23, vertical line a), educational differences across all three deprivation categories were marginal in districts with low and moderate socioeconomic deprivation levels. Around this time, the estimated vaccination rates for individuals with high and medium education levels in all deprivation categories exceeded 0.75, indicating that 75% of this population segment had received at least one vaccine dose. Nevertheless, in highly deprived areas, vaccination rates among people with a low education level had already fallen below this threshold before week 23. Similarly, individuals with low education levels in moderately deprived areas began lagging behind slightly even before the prioritization scheme was lifted. The estimated vaccination rate for individuals with low education levels were decisively lower in highly deprived areas as they had only just surpassed 0.5 by week 27, albeit with significant growth in the subsequent weeks. A comparable trend was observed in regions with moderate deprivation, although the disparity between medium and high education levels was less pronounced than in highly deprived areas.

The nationwide implementation of the 3G rule (week 33, vertical line b) led to another surge in vaccine uptake among people with low education levels residing in highly deprived areas. Estimated cumulative vaccination rates had mostly plateaued for all socioeconomic groups by November 8 (week 45, vertical line c). In areas with high deprivation, educational disparities persisted until then, but these gaps narrowed with the onset of censoring. In regions with moderate deprivation, the gap between people with low education versus medium and high education levels remained largely consistent.

Overall, the reverse survival curves indicate that socioeconomic discrepancies in vaccination rates were evident from early stages of the campaign and amplified after the prioritization scheme was removed. These differences, however, levelled off as the campaign progressed and were much smaller at the start of fieldwork. The temporal dynamics of vaccine uptake showed varying differences, decreasing notably until the November 8. In the multilevel analysis, which follows, we considered three different time points to account for these dynamics.

## Regression analysis with cross-level interactions

Logistic regression models with random intercepts for German districts were used to adjust for confounders and test cross-level interactions. Table 3 presents adjusted odds ratios for the main independent variables and their interactions. Intraclass correlation coefficients (ICC) of the models indicate that considerable variance is found at the district level. Models without cross-level interactions show significant differences in vaccine uptake by education at all three time points (see Models 1a, 2a, and 3a in Table 1). Respondents with lower education levels had lower odds of vaccination compared to those with higher education. A similar but weaker gradient was observed for area-level socioeconomic deprivation, with higher odds of vaccination in less deprived areas.

The coefficients of the interaction terms in Models 1b, 2b and 3b indicate that educational differences varied by socioeconomic deprivation with lower odds of vaccination for low vs. highly educated individuals in moderate and highly deprived areas compared to low vs. highly educated individuals in low deprivation areas at the start of fieldwork. The pattern was similar for all time points, although not all interactions involving groups with low education levels were statistically significant at α = 0.05. In highly deprived areas, the coefficients for those with a medium education level also showed lower odds compared to the group with a high education level with the exception of the second time point. At that date, however, the main effect (adjusted odds ratio (aOR) = 0.549) for the medium vs. highly educated was strongest, indicating substantial differences across all socioeconomic contexts. Otherwise, the interaction effects for a medium education level depending on deprivation were not statistically significant at all time points but consistently ranked between the coefficients for highly deprived areas and 1.

**Table 3:** Adjusted odds-ratios of having received at least one COVID-19 vaccine dose from multilevel logistic regression (p-values in parentheses: + *p* < 0.1, * *p* < 0.05, ** *p* < 0.01, *** *p* < 0.001; adjusted for age, sex, migration history, preexisting medical condition, previous SARS-CoV-2 infection, East/West Germany), N = number of observation (unweighted), ICC = intra-class correlation.

Time point	Prioritization removal		3G Rule		Field start	
	Model 1a	Model 1b	Model 2a	Model 2b	Model 3a	Model 3b
Low Education (Ref. High Education)	0.442^***^ (0.000)	0.617^*^ (0.033)	0.292^***^ (0.000)	0.668 (0.385)	0.371^**^ (0.001)	1.225 (0.630)
Medium Education (Ref. High Education)	0.641^***^ (0.000)	0.766^+^ (0.090)	0.520^***^ (0.000)	0.549^*^ (0.042)	0.574^**^ (0.002)	0.734 (0.323)
High Deprivation (Ref. Low Depriv.)	0.822^+^ (0.095)	1.263 (0.366)	0.671^*^ (0.028)	0.820 (0.591)	0.632^*^ (0.026)	1.279 (0.565)
Moderate Deprivation (Ref. Low Depriv.)	0.722^*^ (0.021)	1.004 (0.982)	0.468^***^ (0.001)	1.003 (0.992)	0.460^**^ (0.003)	1.102 (0.772)
Low Education * High Deprivation		0.356^*^ (0.013)		0.313^+^ (0.069)		0.193^**^ (0.009)
Low Education * Moderate Deprivation		0.739 (0.303)		0.386^+^ (0.090)		0.257^*^ (0.017)
Medium Education * High Deprivation		0.628 (0.132)		1.190 (0.694)		0.663 (0.384)
Medium Education * Moderate Deprivation		0.816 (0.293)		0.871 (0.700)		0.733 (0.424)
Variance (District)	1.583^***^ (0.000)	1.554^***^ (0.000)	3.002^***^ (0.000)	2.580^***^ (0.000)	4.295^***^ (0.000)	3.466^***^ (0.000)
N (individual level)	9,671	9,671	9,671	9,671	9,671	9,671
N (district level)	397	397	397	397	397	397
ICC	0.32	0.32	0.48	0.44	0.57	0.51

**Fig. 2:**
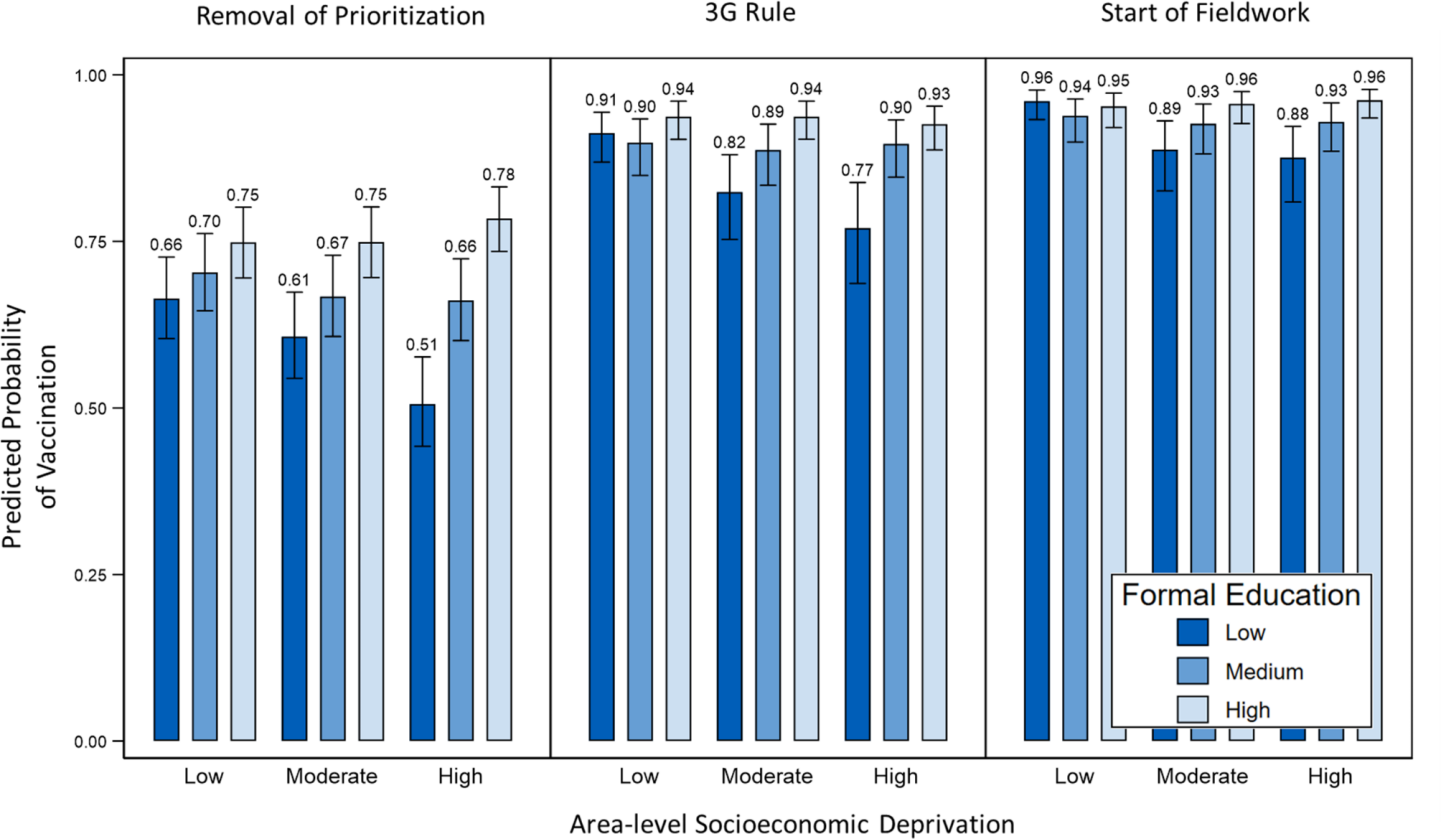
Predicted probabilities for having received at least 1 vaccine dose at three time points (with 95% confidence intervals, based on Models 1b, 2b, and 3b of Table  3).

 For a better understanding of what follows from the interaction effects, average predicted probabilities (i.e., predictive margins) based on Models 1b, 2b, and 3b of Table 3 are shown in Fig. 2. The predicted probability for low-educated individuals dropped with increasing deprivation at all time points. No substantial or significant differences among highly educated people across different deprivation levels were observed. At the removal of the prioritization scheme, an educational gradient was evident for all deprivation levels which led to the highest difference of 27% points between the high and low education level groups in highly deprived areas (p_1_ < 0.001; p_36_ < 0.001). This gradient persisted, though with smaller differences, at the second time point (3G rule) for moderate and high deprivation areas but disappeared in low deprivation areas. By the start of fieldwork, vaccination rates neared or exceeded 90% across all socioeconomic categories, yet differences between high and low education levels persisted in moderate and high deprivation areas. However, these differences were only significant by conventional testing, but not when adjusted for multiple testing (p_1_ < 0.004; p_36_ = 0.094).

## Discussion

### Summary

The present article analyzed educational differences in COVID-19 vaccination status over time and across area-level socioeconomic contexts in Germany. Confirming previous findings, we observed general educational differences in vaccine uptake. However, in areas with low deprivation, where highest vaccination rates were observed, no substantial educational differences in vaccine uptake were found. With increasing levels of area-level socioeconomic deprivation, educational differences were larger due to particularly low vaccination rates in groups with low education levels. People with medium education levels typically had vaccination rates between those with high and low education levels. Consequently, low-educated residents in socioeconomically deprived districts had least likely been vaccinated and therefore faced heightened vulnerability during Germany’s 4th wave of SARS-CoV-2 infections in Fall 2021. Consequently, people in this group may have been particularly vulnerable and that an earlier immunization could potentially have enhanced population immunity.

This is the first study to examine both individual- and area-level socioeconomic gradients in vaccine uptake over the course of Germany’s COVID-19 vaccination campaign. At the individual level, we replicated prior findings that found socioeconomic differences in vaccine uptake in Germany^8^ and confirmed projections of studies that found lower willingness and greater hesitancy to vaccinate for people with lower socioeconomic positions^37–40^. Area-level correlations between socioeconomic conditions and vaccination were also observed^4^. In general, considerable differences between German federal states have been reported based on official notification data, with western and northern states reporting higher rates compared to their eastern and southern counterparts^23,41^. Despite 30 years since German Reunification, economic disparities persist, with East Germany still lagging behind West Germany^42^. Accordingly, a good deal of the socioeconomic differences at the area level were captured in our models that controlled for differences between East and West Germany (see Supplementary Tables C-E). Further research should explore potential confounding of socioeconomic with cultural factors, as East and West Germany also differ in political behavior and trust in institutions^43^. Additionally, variations in vaccination organization across German federal states could have contributed to regional differences in vaccine uptake^44^, suggesting that strategies and their effectiveness may have been correlated with levels of socioeconomic deprivation.

Our investigation of the timing of vaccination revealed a delay in vaccine uptake particular in groups with low education levels, further prolonged in areas of higher levels of socioeconomic deprivation. These educational differences emerged early in the vaccination campaign, notably pronounced in regions with high socioeconomic deprivation, where they only partly reconverged until November. These dynamics confirm concerns voiced by Rydland, et al.^18^, suggesting that individuals with lower socioeconomic positions were less successful in accessing vaccines, particularly when supplies and vaccination opportunities were limited in the campaign’s initial stages. In highly deprived areas, significantly lower vaccination rates were reported by the people with low education until the removal of the prioritization scheme. The percentage point gaps narrowed until the subsequent time point and diminished further by the start of fieldwork, coinciding with widespread vaccine availability and appointment opportunities. Each time point marked different levels of constraints, reflecting the scarcity of vaccines in the early stages and later challenges associated with appointment availability following the removal of prioritization schemes.

Lower vaccination rates among individuals with lower education levels are often attributed to misinformation, which undermines confidence in vaccines — a strong antecedent of vaccination decisions^45^. Studies have shown that individuals with low education levels are more likely to hold misinformed beliefs about COVID-19 vaccines^46^. Misinformation about COVID-19 in general was particularly widespread across social media^47–49^, which was frequently the sole source of COVID-19-related information^50^. Misinformation concerned vaccine safety and efficacy, as well as individuals’ risks through COVID-19 infection^45^. Since individuals with lower education levels in Germany exhibit lower risk perception regarding SARS-CoV-2 infection and a diminished sense of need for protective behavior concerning COVID-19^51,52^, addressing misinformation and improving outreach to groups with lower education levels is crucial for public health interventions.

To our knowledge, no studies have addressed how the area-level socioeconomic context could amplify educational differences in vaccine uptake. Research suggests that perceptions of others’ vaccination behaviors influence one’s own willingness to vaccinate^53,54^, potentially explaining such disparities. Findings from the US reveal that the impact of perceived vaccination behaviors extends beyond close contacts (friends, family, and neighbors) to encompass local (city) and regional (state) levels^54^. Consequently, individual attitudes toward vaccination and corresponding behaviors often mirror perceptions of the local social milieu, particularly among similar peers^55^. Theoretical frameworks on the diffusion of health behaviors emphasize the influence of individuals within the same community, who typically share similar socioeconomic backgrounds and educational levels regarding health behaviors^56^. Consistently, our study illustrates that area-level socioeconomic deprivation patterns impact vaccine uptake and its timing.

### Strengths and limitations

We used data from a scientifically renowned panel study which usually provides a meaningful and accurate reflection of the German population aged 18 and above residing in private households. We applied suitable modeling techniques (multilevel models) to account for the contextual nesting of observations in German districts. Utilizing a categorical classification of area-level socioeconomic deprivation enabled us to uncover nonlinear patterns and to identify individuals with low education levels in highly deprived areas as a group at particularly high risk.

Despite its merits, the study has several limitations. Analyses stratified by regional variables often lack sufficient sample sizes, as does the present study. Thus, the support of statistical significance for the reported educational differences was partly limited to the difference between high- and low-educated individuals. Moreover, the high vaccination rates within the sample exacerbated this statistical power issue, leading to increased standard errors for the differences, despite otherwise adequate sample sizes^57^. Nonetheless, the additional coefficients and predictions suggested further differences including moderate socioeconomic comparison groups, which indicate a gradient of educational level by the level of area-level socioeconomic deprivation. The power problems also kept us from in-depth analyzes of populations at risk. For example, the population aged 60 and older showed already vaccination rates of about 90% by the first time point in our sample (Supplementary Figure C). Due to the prioritization scheme and the much higher overall willingness to vaccinate in these age groups it was unexpected to find meaningful results. Hence, we decided to look at the general adult population including the middling age group which strongly influences the found patterns for the whole adult population (Supplementary Figure B).

Our findings need to be understood in light of the set of adjustment variables. We adjusted for age groups, comorbidities, and migration background, all of which are correlated with educational level. To maintain simplicity for descriptive purposes, we limited further adjustments. As a result, the predicted probabilities for socioeconomic groups from the multiple regression align closely with the descriptive analysis. However, including further covariates, e.g. income levels, would alter educational differences while providing further insights into underlying mechanisms of inequalities in vaccine uptake.

As a further remark, the vaccination rates in the sample (92.7% at the start of fieldwork) systematically exceeded the numbers by official notification data (80.5%, Robert Koch Institute ^6^). While some of this discrepancy could be attributed to time lags in official data, we suspect that respondents with lower education levels and generally lower socioeconomic statuses were underrepresented in our sample—a common problem in survey research, which statistical weighting schemes could not make up for entirely. In turn, if the strength of anti-vaccination attitudes depends on the level of education and is inversely associated with survey participation, educational differences may well have been underestimated in our analysis.

Another caveat concerns the reliance on self-reported vaccination dates, which may be susceptible to recall bias. We contend as panel respondents, participants of GSOEP are accustomed to providing detailed reports on their income, taxes and social security. Checking the date on the vaccination certificate, a document regularly used during the fieldwork phase, would have been a straightforward task. Accordingly, the overall vaccination progress in our data was highly correlated with the officially reported vaccination progress in Germany^23^.

### Conclusion and outlook

Inequity in vaccination coverage is a highly relevant public health issue. The findings can spur future vaccination campaigns to target particular groups and inform initiatives to refine and develop pandemic preparedness planning. As our study delved into novel areas by examining spatial and temporal variations in vaccine uptake, we lacked a comprehensive body of literature on the specific causal mechanisms through which educational differences evolve and why they depend on the socioeconomic context. The attribution and deeper understanding of concrete mechanisms could inform strategies to reach disadvantaged groups and tailor vaccination campaigns more effectively. Existing literature offers insights into strategies to increase vaccine uptake and counter vaccine hesitancy, i.e. stress the importance of individual vaccination for community immunity (‘herd immunity’) or promoting vaccination safety in information campaigns^58,59^. However, little is known about differential effects on particular target groups. Our study suggests that future research and intervention planning should tailor interventions for socioeconomic groups and contexts. The specific targeting of groups with low vaccination rates and high risks of severe illness and death, i.e., groups with low socioeconomic positions, could enhance the effectiveness of vaccination campaigns in future pandemics and mitigate socioeconomic disparities in severe illness and mortality.

## Electronic supplementary material

Below is the link to the electronic supplementary material.

Supplementary Material 1

## Data Availability

The datasets generated and/or analysed during the current study are not publicly available due to German data protection law but are available from the corresponding author on reasonable request. Scripts with the corresponding Stata code used for the empirical analysis can be found in the following repository: https://osf.io/px59v/.
