# Supplementary material for: Reconsidering inequalities in COVID-19 vaccine uptake in Germany: a spatiotemporal analysis combining individual educational level and area-level socioeconomic deprivation
Source: Sci Rep. 2024 Oct 13;14:23904. doi: 10.1038/s41598-024-75273-9 (PMC11471867; doi:10.1038/s41598-024-75273-9)

Supplementary Information

Supplementary Table A: Pandemic Phases and Relevant Events

Pandemic Phase (Variant of Concern)	Duration/ Event	Start	End	7-Day Incidence	Vaccination Rate
Second Wave	Duration	28/09/2020	22/02/2021	max. 204	-
	Vaccination Start	28/12/2020		167	0%
Third Wave (VOC Alpha)	Duration	01/03/2021	13/06/2021	max. 174	-
	Prioritization Removal	07/06/2021		25	55.6%
"Summer Wave" (VOC Delta)	Duration	02/08/2021	04/10/2021	max. 90	-
	3G Rule announced	16/08/2021		39	63.2%
Fourth Wave (VOC Delta)	Duration	05/10/2021	26/12/2021	max. 484	-
	Field start	08/11/2021		219	80.5%
Fifth Wave (VOC Omikron)	Duration	27/12/2021	29/05/2022	max. 1962	-

Sources: Pandemic Phases (Tolksdorf et al. 2022, https://doi.org/10.25646/10598), 7-Day Incidence (Robert Koch-Institut 2024, https://doi.org/10.5281/zenodo.13770938), Vaccination Rate (Robert Koch-Institut 2023, https://doi.org/10.5281/zenodo.7567636)

Supplementary Table B: Variables and Definitions

Construct	Variable	Question	Characteristics	Reference
Vaccine Uptake	One vaccine shot until…<br>…prioritization removal (June 7th)<br>… 3G rule (August 16th)<br>… start of fieldwork (November 8th)	*"Have you been vaccinated against the coronavirus disease (Covid-19)?"*<br>*"Which vaccination did you receive?"*<br>*"Please indicate the date and vaccine agent for each vaccination."*	0 – not vaccinated<br>1 – at least one vaccine shot until the respective time point	Bartig et al. (2022)
Individual Sociodemo-graphic Characteristics	Age Group	based on age calculated by birthyear - year of survey	1 – 18-29 years<br>2 – 30-44 years<br>3 – 45-59 years<br>4 – 60-79 years<br>5 – 80+ years	-
	Sex		1 – male, 2 – female	-
	Migration History (MH)	based on individual life course information	1 – no MH<br>2 – direct MH<br>3 – indirect MH	-
	Education	condensed based on ISCED-11	1 – low education<br>2 – medium education<br>3 – high education	UNESCO Statistics (2012)
Area-level Variables	German Index of Socioeconomic Deprivation (GISD)	based on Quintiles of GISD scores (1 – 1st Quintile, 2-4th Quintile, 3 – 5th Quintile)	1 – low deprivation<br>2 – moderate deprivation<br>3 – high deprivation	Michalski et al. (2022a)
	Survey Region	region based on household residency	1 – West Germany<br>2 – East Germany	-
	District Identifiers	based on household address		-
Chronical Diseases		Grouped count data based on a self-reported checks of a pre-defined list diseases and illnesses	1 – no chronical disease<br>2 – one preexisting condition<br>2 – two or more preexisting conditions	-
Infection with COVID-19	Infected until prioritization removal	*"Have you ever had an infection with the coronavirus (SARS-CoV-2) detected by a PCR test?"*<br><br>*"Date of the (first) positive test:"*	0 – no infection<br>1 – infected until respective time point	Bartig et al. (2022)
	Infected until 3G rule			
	Infected until start of fieldwork			

Supplementary Table C: Odds-Ratios of multilevel logistic regression models at removal of prioritization

	Model 1		Model 2		Model 3		Model 4		Model 5	
Age 18-29 (Ref.)										
Age 30-44	1.041	(0.738)	1.042	(0.730)	1.048	(0.689)	1.059	(0.629)	1.059	(0.625)
Age 45-59	1.974***	(0.000)	1.978***	(0.000)	1.980***	(0.000)	1.976***	(0.000)	1.981***	(0.000)
Age 60-79	6.160***	(0.000)	6.166***	(0.000)	6.194***	(0.000)	6.137***	(0.000)	6.128***	(0.000)
Age 80+	14.32***	(0.000)	14.31***	(0.000)	14.36***	(0.000)	14.16***	(0.000)	14.07***	(0.000)
Male (Ref.)										
Female	1.098	(0.196)	1.097	(0.201)	1.097	(0.202)	1.095	(0.210)	1.096	(0.208)
No migration history (Ref.)										
Direct migration history	0.464***	(0.000)	0.462***	(0.000)	0.457***	(0.000)	0.458***	(0.000)	0.455***	(0.000)
Indirect migration history	0.760	(0.118)	0.756	(0.112)	0.746	(0.096)	0.743	(0.092)	0.743	(0.090)
No pre-existing condition (Ref.)										
One pre-existing condition	1.018	(0.845)	1.020	(0.833)	1.020	(0.832)	1.023	(0.808)	1.026	(0.781)
At least two pre-existing conditions	1.298**	(0.008)	1.299**	(0.008)	1.295**	(0.009)	1.295**	(0.008)	1.293**	(0.009)
No previous SARS-CoV-2 infection (Ref.)										
At least one previous SARS-CoV-2 Infection	0.337***	(0.000)	0.338***	(0.000)	0.341***	(0.000)	0.337***	(0.000)	0.332***	(0.000)
High Education (Ref.)										
Low Education	0.438***	(0.000)	0.442***	(0.000)	0.440***	(0.000)	0.617*	(0.033)	0.620*	(0.035)
Medium Education	0.637***	(0.000)	0.641***	(0.000)	0.641***	(0.000)	0.766	(0.090)	0.771	(0.096)
Low Deprivation (Ref.)										
Moderate Deprivation			0.822	(0.095)	0.879	(0.281)	1.004	(0.982)	0.935	(0.690)
High Deprivation			0.722*	(0.021)	0.857	(0.303)	1.263	(0.366)	1.122	(0.670)
West Germany (Ref.)										
East Germany (incl. Berlin)					0.665***	(0.000)	0.660***	(0.000)	0.884	(0.477)
Low Education # Moderate Deprivation							0.739	(0.303)	0.777	(0.402)
Low Education # High Deprivation							0.356*	(0.013)	0.387*	(0.025)
Medium Education # Moderate Deprivation							0.816	(0.293)	0.914	(0.654)
Medium Education # High Deprivation							0.628	(0.132)	0.759	(0.399)
East Germany (incl. Berlin) # Low Education									0.868	(0.710)
East Germany (incl. Berlin) # Medium Education									0.630*	(0.027)
Var (District)	1.608***	(0.000)	1.583***	(0.000)	1.550***	(0.000)	1.554***	(0.000)	1.545***	(0.000)
N (individual level)	9,671		9,671		9,671		9,671		9,671	
N (district level)	397		397		397		397		397	
ICC	0.32		0.32		0.32		0.32		0.32	

Note: p-values in parentheses: * $p < 0.05$, ** $p < 0.01$, *** $p < 0.001$

Supplementary Table D: Odds-Ratios of multilevel logistic regression models at announcement of 3G rule

	Model 1		Model 2		Model 3		Model 4		Model 5	
Age 18-29 (Ref.)										
Age 30-44	0.494***	(0.000)	0.495***	(0.000)	0.502***	(0.000)	0.505***	(0.000)	0.506***	(0.000)
Age 45-59	1.200	(0.389)	1.205	(0.378)	1.203	(0.382)	1.205	(0.375)	1.209	(0.363)
Age 60-79	2.635***	(0.000)	2.639***	(0.000)	2.660***	(0.000)	2.619***	(0.000)	2.595***	(0.000)
Age 80+	3.038**	(0.004)	3.030**	(0.004)	3.024**	(0.004)	3.006**	(0.005)	2.977**	(0.005)
Male (Ref.)										
Female	0.964	(0.727)	0.962	(0.710)	0.964	(0.727)	0.970	(0.768)	0.971	(0.779)
No migration history (Ref.)										
Direct migration history	0.400***	(0.000)	0.394***	(0.000)	0.382***	(0.000)	0.376***	(0.000)	0.372***	(0.000)
Indirect migration history	0.718	(0.239)	0.712	(0.228)	0.683	(0.171)	0.682	(0.173)	0.684	(0.163)
No pre-existing condition (Ref.)										
One pre-existing condition	0.994	(0.961)	0.999	(0.995)	0.999	(0.994)	1.000	(1.000)	1.004	(0.977)
At least two pre-existing conditions	1.119	(0.419)	1.122	(0.406)	1.118	(0.422)	1.108	(0.460)	1.103	(0.482)
No previous SARS-CoV-2 infection (Ref.)										
At least one previous SARS-CoV-2 Infection	0.232***	(0.000)	0.232***	(0.000)	0.237***	(0.000)	0.235***	(0.000)	0.230***	(0.000)
High Education (Ref.)										
Low Education	0.286***	(0.000)	0.292***	(0.000)	0.290***	(0.000)	0.668	(0.385)	0.677	(0.400)
Medium Education	0.514***	(0.000)	0.520***	(0.000)	0.521***	(0.000)	0.549*	(0.042)	0.556*	(0.046)
Low Deprivation (Ref.)										
Moderate Deprivation			0.671*	(0.028)	0.827	(0.303)	1.003	(0.992)	0.864	(0.618)
High Deprivation			0.468***	(0.001)	0.778	(0.285)	0.820	(0.591)	0.646	(0.254)
West Germany (Ref.)										
East Germany (incl. Berlin)					0.333***	(0.000)	0.327***	(0.000)	0.526*	(0.036)
Low Education # Moderate Deprivation							0.386	(0.090)	0.455	(0.142)
Low Education # High Deprivation							0.313	(0.069)	0.401	(0.197)
Medium Education # Moderate Deprivation							0.871	(0.700)	1.074	(0.844)
Medium Education # High Deprivation							1.190	(0.694)	1.659	(0.282)
East Germany (incl. Berlin) # Low Education									0.597	(0.409)
East Germany (incl. Berlin) # Medium Education									0.518	(0.055)
Var (District)	3.233***	(0.000)	3.002***	(0.000)	2.544***	(0.000)	2.580***	(0.000)	2.542***	(0.000)
N (individual level)	9,671		9,671		9,671		9,671		9,671	
N (district level)	397		397		397		397		397	
ICC	0.50		0.48		0.44		0.44		0.44	

Note: p-values in parentheses: * $p < 0.05$, ** $p < 0.01$, *** $p < 0.001$

Supplementary Table E: Odds-Ratios of multilevel logistic regression models at start of fieldwork

	Model 1		Model 2		Model 3		Model 4		Model 5	
Age 18-29 (Ref.)										
Age 30-44	0.594*	(0.014)	0.597*	(0.015)	0.606*	(0.019)	0.615*	(0.022)	0.617*	(0.022)
Age 45-59	1.191	(0.507)	1.195	(0.498)	1.191	(0.506)	1.188	(0.506)	1.194	(0.491)
Age 60-79	2.319**	(0.002)	2.323**	(0.001)	2.342**	(0.001)	2.311**	(0.002)	2.264**	(0.002)
Age 80+	2.097	(0.080)	2.091	(0.082)	2.087	(0.084)	2.058	(0.091)	2.025	(0.099)
Male (Ref.)										
Female	0.970	(0.811)	0.967	(0.792)	0.970	(0.806)	0.972	(0.821)	0.972	(0.818)
No migration history (Ref.)										
Direct migration history	0.465***	(0.000)	0.459***	(0.000)	0.442***	(0.000)	0.434***	(0.000)	0.426***	(0.000)
Indirect migration history	0.568	(0.074)	0.563	(0.069)	0.536*	(0.046)	0.537*	(0.048)	0.540*	(0.039)
No pre-existing condition (Ref.)										
One pre-existing condition	0.979	(0.890)	0.984	(0.917)	0.981	(0.903)	0.981	(0.904)	0.984	(0.918)
At least two pre-existing conditions										
No previous SARS-CoV-2 infection (Ref.)										
At least one previous SARS-CoV-2 Infection	0.397***	(0.000)	0.397***	(0.000)	0.406***	(0.000)	0.403***	(0.000)	0.395***	(0.000)
High Education (Ref.)										
Low Education	0.364***	(0.001)	0.371**	(0.001)	0.369**	(0.001)	1.225	(0.630)	1.250	(0.595)
Medium Education	0.567**	(0.001)	0.574**	(0.002)	0.576**	(0.002)	0.734	(0.323)	0.750	(0.357)
Low Deprivation (Ref.)										
Moderate Deprivation			0.632*	(0.026)	0.807	(0.306)	1.102	(0.772)	0.851	(0.604)
High Deprivation			0.460**	(0.003)	0.825	(0.508)	1.278	(0.565)	0.904	(0.815)
West Germany (Ref.)										
East Germany (incl. Berlin)					0.291***	(0.000)	0.288***	(0.000)	0.616	(0.160)
Low Education # Moderate Deprivation							0.257*	(0.017)	0.376	(0.062)
Low Education # High Deprivation							0.193**	(0.009)	0.314	(0.120)
Medium Education # Moderate Deprivation							0.733	(0.424)	1.034	(0.931)
Medium Education # High Deprivation							0.663	(0.384)	1.036	(0.942)
East Germany (incl. Berlin) # Low Education									0.327	(0.088)
East Germany (incl. Berlin) # Medium Education									0.381**	(0.008)
Var (District)	4.694***	(0.000)	4.295***	(0.000)	3.457***	(0.000)	3.479***	(0.000)	3.466***	(0.000)
N (individual level)	9,671		9,671		9,671		9,671		9,671	
N (district level)	397		397		397		397		397	
ICC	0.59		0.57		0.51		0.51		0.51	

Note: p-values in parentheses: * $p < 0.05$, ** $p < 0.01$, *** $p < 0.001$

Supplementary Figure A: Education-specific reverse Kaplan—Meier survival curves for first vaccination by area-level socioeconomic deprivation over calendar weeks in 2021 (a – Prioritization removal, b – 3G Rule, c – Field start) aged 18 and older, unweighted, with 95% confidence intervals

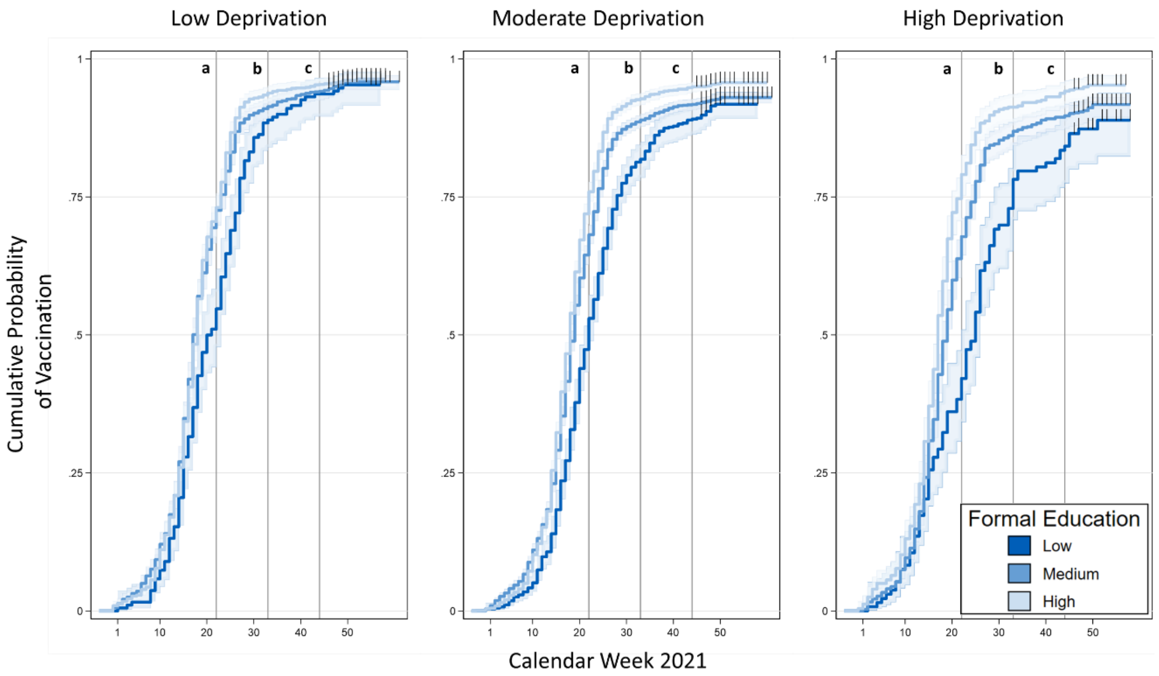

Supplementary Figure B: Education-specific reverse Kaplan—Meier survival curves for first vaccination by area-level socioeconomic deprivation over calendar weeks in 2021 (a – Prioritization removal, b – 3G Rule, c – Field start) aged 30 to below 60, weights applied

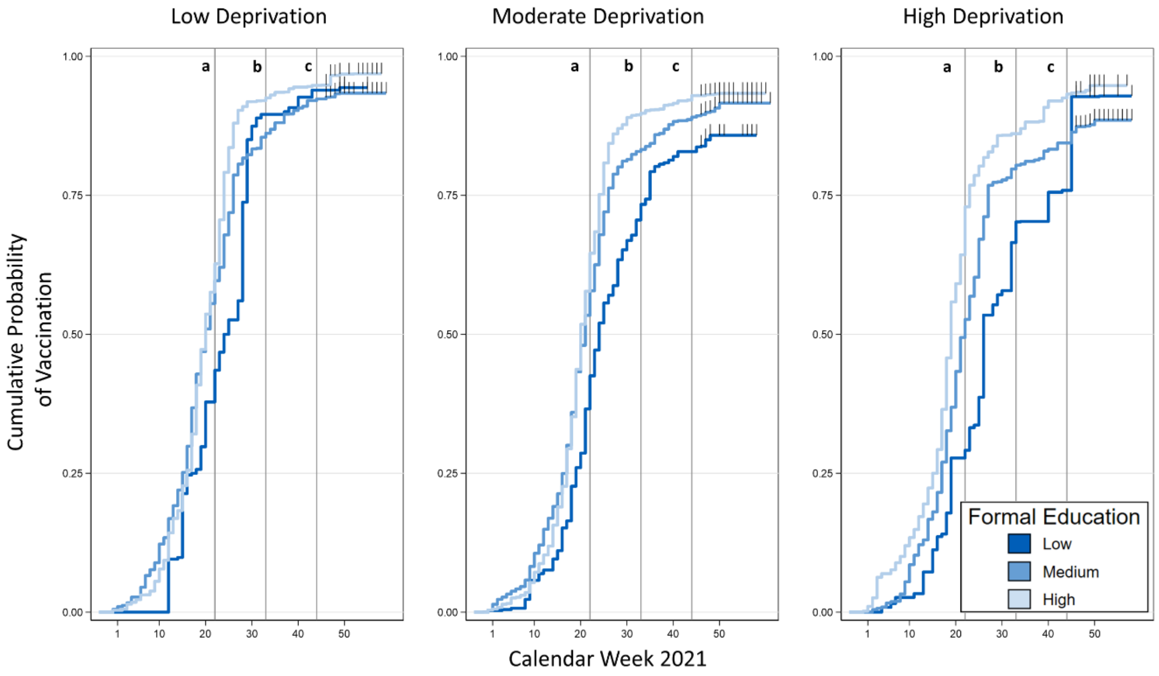

Supplementary Figure C: Education-specific reverse Kaplan—Meier survival curves for first vaccination by area-level socioeconomic deprivation over calendar weeks in 2021 (a – Prioritization removal, b – 3G Rule, c – Field start) aged 60 and older, weights applied

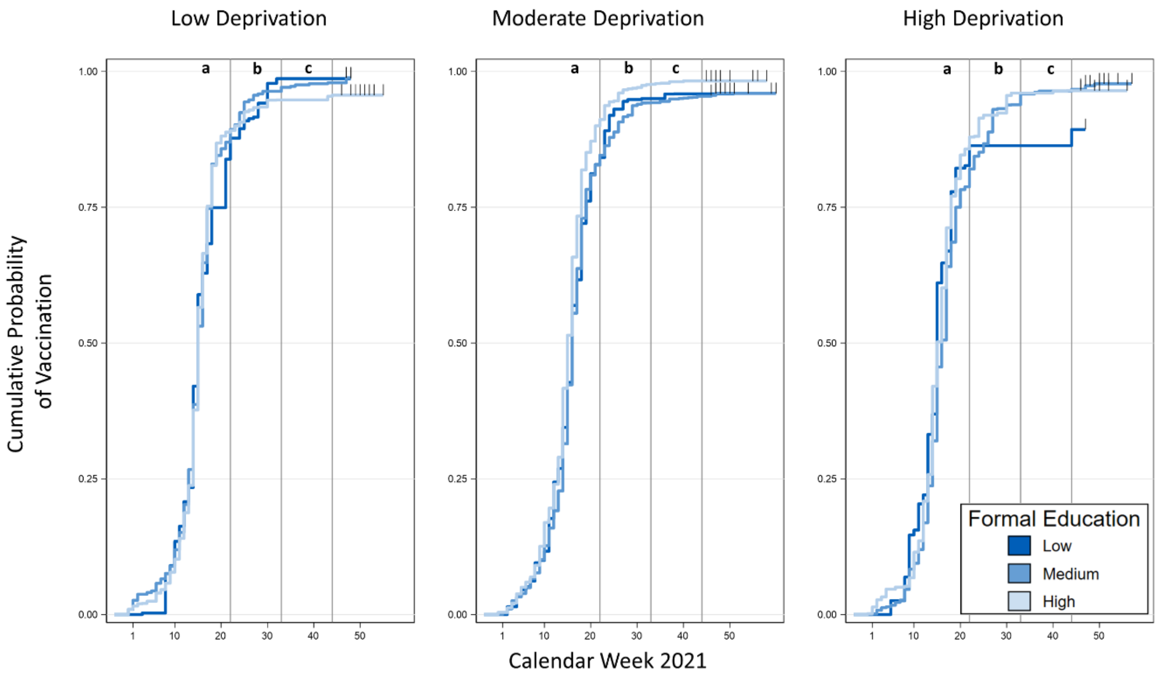

Supplement: Supplementary file 1 — Supplementary Material 1 [file 41598_2024_75273_MOESM1_ESM.pdf]
